# Root systems biology

**DOI:** 10.3389/fpls.2014.00215

**Published:** 2014-05-19

**Authors:** Wolfgang Schmidt

**Affiliations:** Institute of Plant and Microbial Biology, Academia SinicaTaipei, Taiwan

**Keywords:** root architecture, root hairs, systems biology, synthetic biology, auxin, regulatory peptides, nutrient acquisition, gene co-expression analysis

Plant roots, which are essential for providing anchorage to the soil, acquiring mineral nutrients and water, and for synthesizing a plethora of metabolites, provide an excellent model for studying physiological, developmental, and metabolic processes at a systems level. The challenge to understand such processes has been compared with deciphering the principle of a radio by a reductionist approach, i.e., by randomly removing parts from a series of identical radios and observing the “phenotypes” resulting from this procedure (Lazebnik, 2002). Undoubtably, understanding (root) biology as a whole represents a much bigger challenge, but the constant development of novel tools and algorithms as well as technical progress on omics technologies facilitate rapid progress toward a more integrative, holistic picture of root biology. The 13 articles in this ebook highlight the latest results, approaches, and resources in root systems biology.

One challenge when studying roots is their multicellular complexity. Qiao and Libault (2013) describe a method in which an ultrasound aeroponic system is employed to generate a large quantity of root hair cells, allowing for an uniform and long-term treatment of a single cell type with various biotic and abiotic stimuli for downstream functional genomics applications. Root hair development is affected by soil environmental factors that maximize the absorption capacity and, ultimately, the fitness of the plant. Lee and Cho (2013) summarize the role of auxin as a key player and organizing node for environmental/hormonal modulation of root hair growth. Auxin plays also a key role in the formation of lateral roots which, post-embryonically initiated from the primary root in response to developmental and environmental stimuli, provide a high level of plasticity to the root system architecture. New generation imaging techniques and high-throughput approaches, often used in combination with computational modeling, have triggered a revival of root development research. In their review article, Cuesta et al. (2013) describe traditional and novel tools, and evaluate their potential to address longstanding questions on lateral root organogenesis at a qualitatively new level.

Root architecture is closely interconnected with and shaped by the availability of nutrients, in particular nitrate and phosphate. Strategies for enhanced resource acquisition in crops are of increasing importance to secure sustainable food production. Such strategies have recently focused on root traits with the aim of a more efficient utilization of soil resources that would facilitate the transition from high-input monoculture-based agriculture to productive, sustainable agro-ecosystems with low inputs. Tian and Doerner (2013) evaluate the importance of root resource foraging and the possibility of exploiting natural variants in landraces or wild relatives of crops for breeding programs with the aim of producing crops with root traits that allow for a more resilient performance when experiencing environmental stresses such as phosphate deficiency. Nitrogen, mainly taken up as nitrate, is another essential nutrient that strongly affects root architecture and is critical for plant productivity. The modulation of root development by N availability has great agricultural importance and its understanding provides the basis for the generation of germplasms with improved root architecture. Mohd-Radzman et al. (2013) provide an update of the current knowledge of the signaling components involved in N-mediated root architecture, giving special emphasis on the legume root system. Deficiency of nitrate results in the expression of approximately 2000 genes from which only a minority has yet been functionally characterized. By integrating publicly available microarray data from 27 independent nitrate-related experimental datasets, Canales et al. (2014) generated several highly co-expressed gene clusters with robust functions in nitrate transport, signaling, and metabolism in *Arabidopsis* roots. In addition to prioritizing potentially important genes for further functional characterization, the meta-analysis uncovered several putative key regulatory factors that control these gene network modules and highlight novel nitrate-controlled developmental processes such as root hair formation.

The transition zone of the root connects the highly sensitive root apex with the elongation zone in which responses to environmental stimuli are accomplished, resulting in changes in cell fate and alterations in root architecture. Baluška and Mancuso (2013) discuss the specific features of the transition zone and hypothesize that it acts as a command zone that integrates environmental information received from the apex to regulate responses of cells in the elongation zone. Abiotic stress such as drought, salinity, flooding, and cold adversely affect plant growth and decline crop productivity. Stressor-specific protein signatures that dictate adaptive mechanisms are described from a proteomics perspective by Ghosh and Xu (2014). Advances in mass spectrometry and peptide fragmentation dramatically improve the coverage of proteomic profiles and opens up new perspectives for the dissection of molecular mechanisms underlying adaptive responses to abiotic stresses.

Intrinsically disordered proteins do not adopt a folded structure in their functional form, but perform functions of critical importance in signaling cascades and transcription factor networks. Owing to their intrinsic conformational flexibility, disordered proteins can bind multiple partners with high specificity and low affinity, thereby adding complexity to the interactomes. Far from being rare or anecdotal, disordered proteins are among the most important proteins in a given proteome, apparently contradicting the classical structure-function relationship. Pazos et al. (2013) postulate that protein disorder is particularly important for the sessile lifestyle of plants, providing them with a fast mechanism to obtain intricate, interconnected, and versatile molecular networks for interacting with the environment.

More than 7000 small, unannotated open reading frames, many of which may encode regulatory peptides, exist in the *Arabidopsis* genome (Hanada et al., 2013). Small signaling peptides are a growing class of regulatory molecules which are part of the myriad of signaling networks that control the development of plant roots. Delay et al. (2013) review the involvement of regulatory peptides in several aspects of plant root development, including but not limited to meristem maintenance, the gravitropic response, lateral root development and vascular formation, highlighting the recent leap in our understanding of their role in the regulation of developmental programs.

Gene regulatory networks (GRNs) are an excellent tool for the integration and analysis of complex biomolecular systems at the structural and dynamic level. However, most GRN models are incomplete because they likely lack components or interactions due to sketchy experimental data and computational limitations. Azpeitia et al. (2013) propose a set of procedures for detecting and predicting missing interactions in Boolean networks and evaluate their applicability to predict putative missing interactions using a previously published *Arabidopsis* root stem cell nice network as an example (Azpeitia and Alvarez-Buylla, 2012).

Research into root biology has greatly profited from engineering plants to express multi-component DNA constructs such as promoter/reporter gene fusions. Emami et al. (2013) introduce an optimized protocol for the rapid and inexpensive generation of multi-component transgenes based on the Golden Gate cloning strategy. Simultaneous monitoring of membrane potential changes in populations of cells would provide a quantifiable characteristic to evaluate together with global changes in gene activity and metabolite levels in systems biology research. Matzke and Matzke (2013) describe the production of transgenic plants engineered to express different versions of genetically encoded voltage-sensitive fluorescent proteins that are targeted to the plasma membrane and internal membranes of plant cells. Their Hypothesis and Theory article describes progress toward adapting a technology originally used on animal nerve cells to record electrical patterns that transcend single cell boundaries and single membrane systems in response to various stimuli in living plants.

## Conflict of interest statement

The author declares that the research was conducted in the absence of any commercial or financial relationships that could be construed as a potential conflict of interest.
